# The first report of rhinosinusitis by *Rhizopus delemar* in a patient with severe COVID-19 in Iran: a case report

**DOI:** 10.1186/s13256-024-04873-w

**Published:** 2024-11-05

**Authors:** Seyedeh Sabereh Mojtahedi, Neginsadat Hosseinikargar, Hossein Zarrinfar, Mehdi Bakhshaee, Mohammad Javad Najafzadeh, Ya Bin Zhou, Jos Houbraken

**Affiliations:** 1 Student Research Committee, Mashhad University of Medical Sciences, Mashhad, Iran; 2 Department of Parasitology and Mycology, School of Medicine, Mashhad University of Medical Sciences, Mashhad, Iran; 3 Department of Laboratory Sciences, School of Paramedical Sciences, Mashhad University of Medical Sciences, Mashhad, Iran; 4 Allergy Research Center, Mashhad University of Medical Sciences, Mashhad, Iran; 5 Sinus and Surgical Endoscopic Research Center, Department of Otorhinolaryngology, Mashhad University of Medical Sciences, Mashhad, Iran; 6 Department of Dermatology, Beijing Children’s Hospital, Capital Medical University, National Center for Children’s Health, Beijing, China; 7 Westerdijk Fungal Biodiversity Institute, Utrecht, The Netherlands

**Keywords:** Mucormycosis, COVID-19, *Rhizopus delemar*, Rhinosinusitis, Infection

## Abstract

**Background:**

Mucormycosis is a severe and fatal fungal infection in patients with coronavirus disease 2019 caused by *Mucorales*. Here we present a case of a 63-year-old man with coronavirus disease 2019 infection, along with rhinosinusitis mucormycosis caused by *Rhizopus delemar*.

**Case presentation:**

A 63-year-old Iranian man suffering from a coronavirus disease 2019 infection with symptoms of cough, shortness of breath, and generalized body pain. On the basis of the clinical manifestations, such as headache, a history of black nasal discharge, nasal hypoesthesia, facial swelling, numbness, nasal obstruction, presence of necrotic lesions on the nasal passages on physical examination, and abnormal computed tomography scans of paranasal sinuses, the patient underwent surgical debridement. Direct microscopy of specimens obtained from the paranasal sinuses, and subsequent isolation and identification, revealed a rhinosinusitis mucormycosis caused by *R*. *delemar*. Despite therapeutic measures, such as sinus debridement surgery and antifungal therapy with amphotericin B injection (50 mg/day), the patient died after 35 days of hospitalization.

**Conclusion:**

In this report, we present the first documented case of human infection with *R*. *delemar* in a patient with coronavirus disease 2019 infection, who also exhibited rhinosinusitis mucormycosis. *R*. *delemar* appears to be an emerging agent of rhinosinusitis mucormycosis in this region. Furthermore, prompt diagnosis and the exploration of alternative antifungal treatments, beyond amphotericin B, may be crucial for effectively managing patients with *R*. *delemar* infections.

## Introduction

Mucormycosis (previously named zygomycosis) is a life-threatening fungal infection caused by species of the order *Mucorales* that mainly affects immune-deficient individuals, such as those with hematological malignancy, organ transplantation recipients, and people with diabetes [1]. The prevalence of fungal (co) infections, including mucormycosis, increased during the coronavirus disease 2019 (COVID-19) pandemic owing to the widespread use of corticosteroids, broad-spectrum antibiotics, and prolonged hospitalization in hospital wards [2, 3]. The primary species causing mucormycosis belong to *Rhizopus*, *Mucor* and *Lichtheimia* (formerly *Absidia*), and these can invade blood vessels and cause problems, such as inflammation and blood clots [4], resulting in serious damage and death of tissue. *R*. *delemar* is a rare opportunistic pathogen causing invasive and disseminated disease in human (mucormycosis), but it is also common in the environment and can be isolated from for example, decayed plant material and soils. The signs and symptoms of mucormycosis are not specific and therefore *R*. *delemar* infections are difficult to diagnose [5, 6], and need to be identified through a combination of clinical and radiological observations, histopathological procedures, and laboratory criteria [7]. However, some of these methods are time consuming and are not very effective at detecting the infection [5]. This delay in diagnosis means that patients with mucormycosis might not be properly treated on time, resulting in a higher chance of a poor outcome [8]. Here we present a case of a 63-year-old man affected with COVID-19 infection, along with rhinosinusitis mucormycosis caused by *R*. *delemar*.

## Case report

**Fig. 1:**
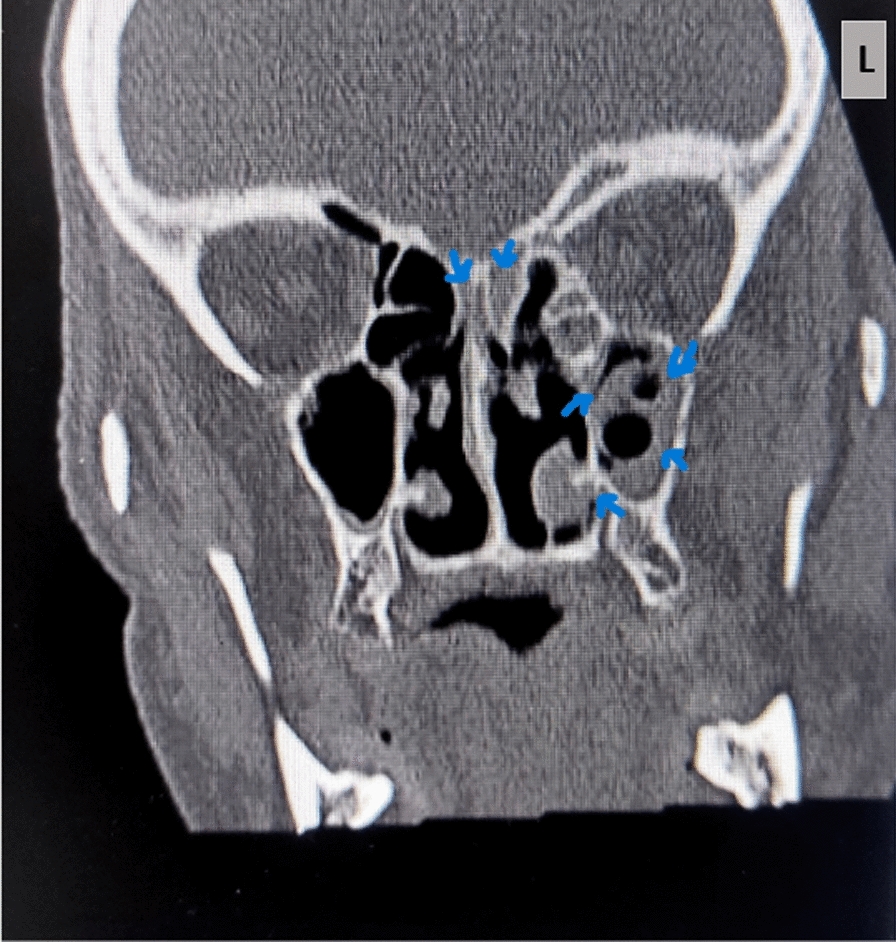
Paranasal sinus computed tomography scan, turbidity, and increased mucus thickness are observed in the maxillary, sphenoid, and frontal sinuses of the right and left ethmoid (arrowhead)

**Fig. 2:**
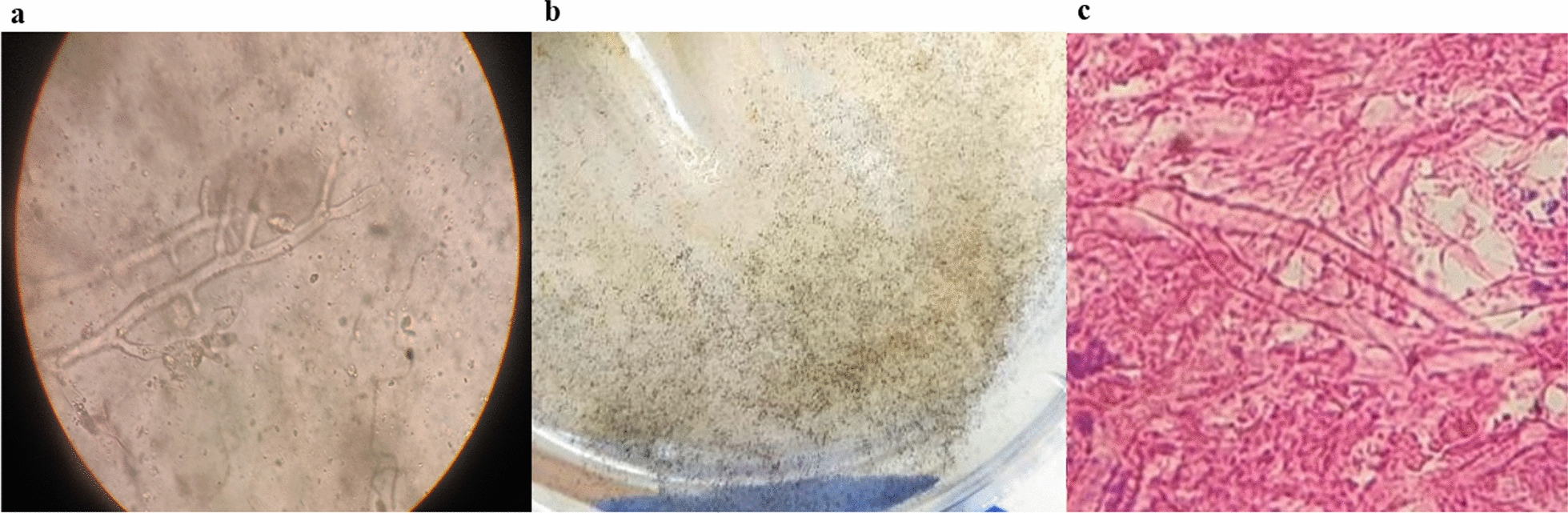
**a** Appearance of broad aseptate hyphae in direct examination (20% KOH staining, original magnification × 400), **b** Appearance *R*. *delemar* colonies on SC, and **c** Non-septate hyphae in histopathological examination (periodic acid-Shiff staining, original magnification × 400)

**Fig. 3:**
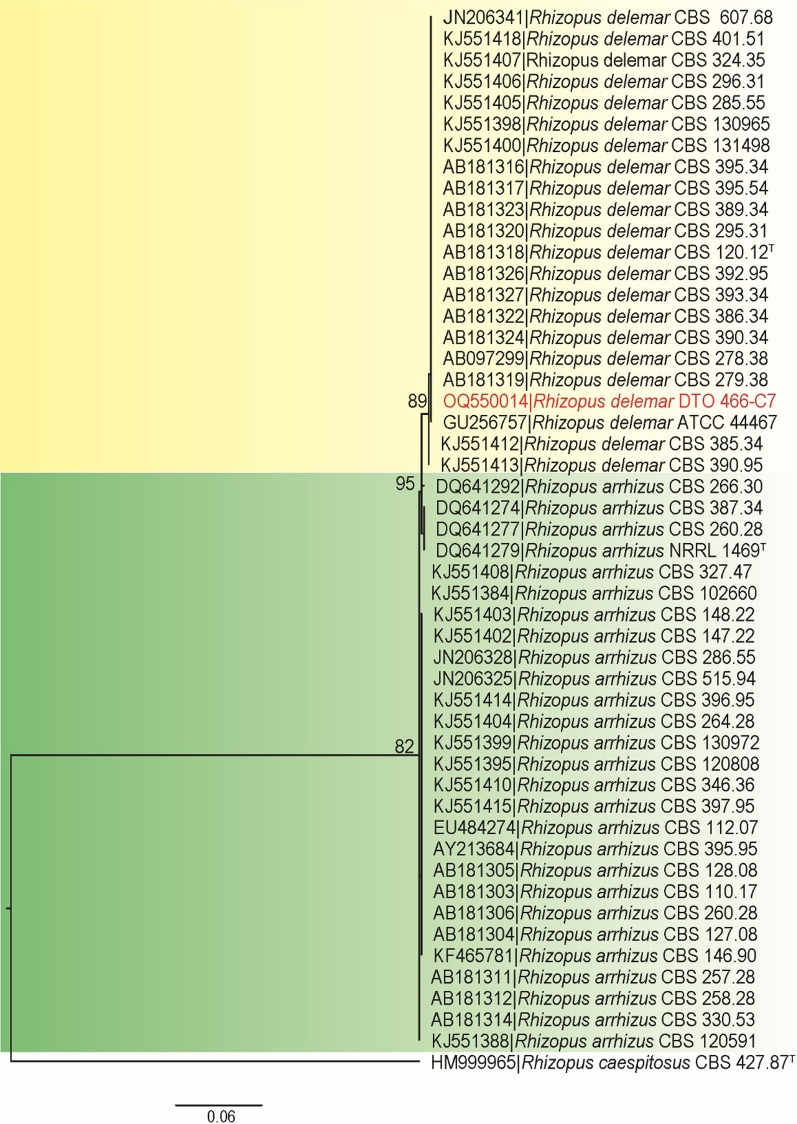
Phylogenetic tree based on internal transcribed spacer sequences. The bootstrap percentages of the maximum likelihood analysis are presented at the nodes. Values with less than 70% bootstrap support (maximum likelihood) are not displayed. The bar indicates the number of substitutions per site. The phylogram is rooted with *R*. *caespitosus*. Tex-type strain

A 63-year-old man living in Mashhad, northeastern Iran, was admitted to Imam Reza Hospital in Mashhad in September 2021. His main complaints were shortness of breath, cough, and generalized body pain. The chest computed tomography (CT) scan revealed bilateral diffuse interstitial opacities. Unfortunately, there are no available reports regarding the patient’s family and psychosocial history, as well as any prior interventions. However, there were no reports of any specific underlying conditions, including diabetes, in this patient. He was admitted to the COVID-19 ward with an initial diagnosis of acute respiratory failure on day 0. To confirm COVID-19, nasopharyngeal and oropharyngeal specimens were retrieved and the presence of the ribosomal RNA (rRNA)-dependent RNA polymerase (RdRp) gene was measured using real-time reverse-transcription polymerase chain reaction (rRT-PCR, Pishtaz Teb, Lot.99006) technique. On the day of admission (day 0), his hematological findings showed white blood cell (WBC) = 9.7 × 10^9^/L [4.1–10 × 10^9^], red blood cell (RBC) = 3.45 × 10^12^/L [4.3–6.2 × 10^12^], hemoglobin (Hb) = 9.8 g/dl [13.2–16.5], hematocrit (Ht) = 29.8% [40–51], platelet (Plt) = 140 × 10^9^/L [140–450 × 10^9^], mean corpuscle (cell) volume (MCV) = 86.4 fl [82–102], mean corpuscular hemoglobin (MCH) = 28.4 pg [27–31], mean corpuscular hemoglobin concentration (MCHC) = 32 g/dl [31–35], red blood cell distribution width (RDW)-CV = 17.4% [11.5–14.5], mean platelet volume (MPV) = 10.6 fl [7.5–12], neutrophil (Neu) = 70% [50- 70], and lymphocyte (Lym) = 20% [25–40]. Administration of prednisolone was started (day + 5) at a dose of 125 mg per day for a duration. Additionally, the patient was prescribed broad-spectrum antibiotics cefepime (day + 5), vancomycin, imipenem (day + 7), and meropenem (day + 12). Blood cultures were taken at day + 10 and they were negative after 48 and 96 h. On the 19th day of admission the patient’s blood sugar (BS) was 194 mg/dl. Table 1 showed the other laboratory investigations. The patient required mechanical ventilation owing to the increased hypoxia and progressive decline in oxygen saturation (day + 17). At 20 days after admission (day + 20), the physicians suspected rhinosinusitis according to physical examination and clinical manifestation, such as headache, facial swelling, numbness, and nasal obstruction. Subsequently, the patient visited otorhinolaryngology and infectious disease specialists. In their investigation, lesions on the nasal passages and necrotic crust were noticed that leaded to computed tomography (CT) investigations. On the paranasal sinus (PNS) CT scan (Fig. 1), turbidity and increased mucus thickness were observed in the maxillary, sphenoid, and frontal sinuses of the right and left ethmoid. The patient underwent sinus debridement to eradicate infection and sinus biopsies were obtained (day + 21). Tissue specimens were sent to the medical mycology and pathology laboratory for further diagnostic work-up. Diagnosis of mucormycosis was made on the basis of a presence of broad aseptate hyphae in 20% potassium hydroxide (KOH) preparation of specimens obtained from the paranasal sinuses (day + 21), and cultivation on Sabouraud’s dextrose agar with chloramphenicol (Merck, Germany) medium at 35 °C for 3 days. While the sensitivity of direct examination and culture methods is known to be quite low, they remain highly valuable because of their cost-effectiveness and accessibility. The isolation plates showed greyish brown colonies that covered an agar surface in 2 days after incubation (day + 23). Moreover, histopathological examination using periodic acid-Shiff (PAS) stained sections showed a dense accumulation of broad and non-septate hyphae (Fig. 2). The isolated fungal strain was identified using phenotypic characters, and sequencing the internal transcribed spacers (ITS) including the 5.8S nrRNA gene region ITS and a part of the large subunit (LSU) 28S rRNA. Macroscopic observations of the strain after 5 days (day + 26) on malt extract agar at 25 °C showed spreading, elevated, grey colored colonies, and microscopic examination of the strain showed unbranched, pigmented sporangiophores with rhizoids, and apophyses, a combination of structures often observed in *Rhizopus* strains. ITS and LSU sequences were generated, following the protocols described in Samson *et al*. (2019) [9]. In short, genomic DNA was extracted using the DNeasy® UltraClean® Microbial Kit (Qiagen, Germany). Amplification and sequencing of the ITS and LSU fragments was performed using the primer pairs V9G & LS266 and LR0R & LR5, respectively. Homology searches (BLAST) with the consensus sequences were performed to identify the strain at species level. The ITS and LSU sequence were identical to sequences generated from the type of *R*. *delemar* (CBS 120.12; ITS, AB181318; LSU, MH866134). The newly generated sequences were deposited in GenBank under accession numbers OQ533696 (LSU) and OQ550014 (ITS), and the strain was deposited in the DTO collection under number DTO 466-C7 (working collection in Food and Indoor Mycology group in Westerdijk Institute). To achieve a more precise identification of our isolate at the species level, we conducted a phylogenetic analysis on the basis of ITS sequences. Figure 3 provides an overview of strains, species, and their corresponding NCBI GenBank accession numbers. *R*. *caespitosus* CBS 427.87^T^ served as the outgroup. The resulting phylogenetic tree (Fig. 3) indicates that strain DTO 466-C7 belongs to the *R*. *delemar* clade with high statistical support. Since the sufficient growth of the strain did not occur during incubation of subcultures, antifungal susceptibility test was not performed. For treatment, amphotericin B injection (50 mg/day) were prescribed (day + 20). On the 19th day of hospitalization, remdesivir was administrated and continued for 7 days. Although amphotericin B and isavuconazole are the two agents approved for the primary treatment of mucormycosis, isavuconazole was not available for prescription. Unfortunately, serial debridement surgeries and endoscopy of the sinus tissues were not possible owing to the patient unstable state. Despite therapeutic measures, no considerable improvement was observed. Unfortunately, the patient died after 35 days of hospitalization (day + 35).

**Table 1:** The laboratory tests results of the patient, on the 19th day of admission

Test	Patient’s result	References interval
AST	87 U/L	5–40
ALT	162 U/L	7–56
ALP	297 U/L	45–148
Na	136 mEq/L	135–145
K	4.2 mEq/L	3.6–5.2
Urea	52 mg/dL	15–45
Cr	0.9 mg/dL	0.9–1.3

*AST* Aspartate aminotransferase, *ALT* Alanine aminotransferase, *ALP* Alkaline phosphatase, *Na* Soduim, *K* Potassium, *Cr* Creatinine

## Discussion

Fungal rhinosinusitis includes a diverse range of fungal infections, varying from mild irritations to those that can quickly become life-threatening [2, 10–12]. The reports of COVID-19-associated RM have been increasing during the COVID-19 pandemic along with high morbidity and mortality, particularly owing to patients with cerebral involvement [2, 11]. Within *Mucorales*, *Rhizopus* and *Mucor* species were the most commonly identified causative agents, and *R*. *arrhizus* has been reported as the predominant causal agent worldwide [8]. The current study presents a novel case of a patient with a COVID-19 infection, along with RM caused by *R*. *delemar*. In SağıtM *et al*., the total number of patients with rhino-orbital mucormycosis (ROM) between 2013 and 2016 was six over 2 years [13]. In the study of Nurettin Bayram *et al*., who conducted their study during the coronavirus outbreak, they estimated the frequency of the disease would be 11 patients among patients with serious COVID-19, during 9 months [14]. According to estimates, mucormycosis affects 1.7 million people every year [15]. In immunocompromised individuals, *R*. *delemar* is an invasive fungal pathogen that has the potential to cause fatal mucormycosis. Early diagnosis is difficult for mucormycosis caused by *R*. *delemar*, which is a rare and difficult condition. Owing to the similarity of *R*. *delemar* to other species, this species has not been reported frequently yet, and the precise diagnosis requires molecular methods. A 47-year-old male with a brainstem hemorrhage caused by *R*. *delemar* was reported by Shuhua Xie *et al*. [6]. The diagnosis of encephalitis caused by *R*. *delemar* was made using metagenomic next-generation sequencing (mNGS) in cerebrospinal fluid (CSF) and the patient’s clinical characteristics [6]. In our report, the obtained sinus biopsy showed the presence of this organism after isolation and subsequent sequencing. Inhalation of *R*. *delemar* spores from the environment is the main way route of exposure, where infections in the lungs and nose affecting the brain are very common [16]. Neutropenia or glucocorticoid therapy is thought to be a risk factor for rhino-cerebral and pulmonary infections, while diabetic patients are more likely to develop rhino-cerebral mucormycosis [17]. People with diabetes, especially those with a severe type called diabetic ketoacidosis, are the most common patients (accounting for 58.9–86.7%) to have a fungal infection affecting their nose, eyes, and brain, called nasal-orbital-cerebral mucormycosis [4] In the study of Nurettin Bayram *et al*., among the 11 patients investigated who were simultaneously affected by COVID-19 and tuberculosis, 8 patients also had diabetes [14]. However, our patient did not have diabetes. Basal ganglia mucormycosis was also found in a case that did not have any known cause of immunosuppression but had a history of drug injection [18]. Even with medication and surgery to treat a fungal infection, there is a high mortality rate within a few months after diagnosis. The mortality rate can range from 50% to 99% depending on the infection severity when treatment starts [19]. On the other hand, mucormycosis has a pronounced tendency to enter blood vessels, causing thrombosis, necrosis, and tissue infarction, all of which have a high mortality rate [20]. In this case, the patient died despite fungal prophylaxis and treatment. The CT scans and magnetic resonance imaging (MRI) can show signs of thickened and darkened sinuses, and involvements in the eyes and brain. These imaging methods helped find seven patients with rhino-orbital mucormycosis and three patients with rhino-orbital-cerebral mucormycosis [14]. In their study, the foremost commonly included sinuses were ethmoid (90.9%) followed by maxillary sinuses (81.8%), which is in understanding with the study of Gupta *et al*. [21]. In our patient, paranasal sinus involvement was also observed on his CT scan. It is worth noting that for a more detailed examination of immunohistochemistry, PCR for fungal DNA, PCR sequencing, and in situ hybridization can be utilized for elective methods of tissue detection [22]. As soon as the diagnosis is suspected, mucormycosis treatment should be initiated according to the patient’s condition. Amphotericin B is recommended as the best medication for treating mucormycosis according to the global guideline and other study findings [23]. In this case, despite the patient receiving amphotericin B, the patient died. Unfortunately, owing to the restrictions created during the COVID-19 pandemic in hospitals, posaconazole or isavuconazole antifungals were not available for prescription. However, it is still not clear whether underlying diseases, fungal species, or coinfection with COVID-19 caused this patient’s lack of successful treatment. The study by Mehta *et al*. has shown that the coexistence of ROM and extreme infection of COVID-19 has a higher mortality rate than already detailed nonCOVID-19 patients [24]. According to these findings, the prognosis of preexisting ROM may worsen after a severe severe acute respiratory syndrome coronavirus 2 (SARS-CoV-2) infection. The primary factors that appear to predispose patients to immune dysregulation associated with SARS-CoV-2 include the widespread use of steroids and broad-spectrum antibiotics, as well as the need for ventilatory support. Therefore, it is important for physicians to be aware of extra potential infections in patients with COVID-19. However, whether these local and systemic factors resulted in any predisposition has not yet been clearly established. Hence, reported infections by *R*. *delemar* in these patients show that other risk factors may also be important in the development of infection.

## Conclusion

RM owing to *R*. *delemar* in critically ill patients with COVID-19 is a potentially lethal complication. It seems likely that faster diagnosis and the use of other antifungal drugs, except amphotericin B, can play a more vital role in treatment of patients with this infection.

## Data Availability

Not applicable.
